# Compartmentalization of anti-oxidant and anti-inflammatory gene expression in current and former smokers with COPD

**DOI:** 10.1186/s12931-019-1164-1

**Published:** 2019-08-20

**Authors:** Venkataramana K. Sidhaye, Janet T. Holbrook, Alyce Burke, Kuladeep R. Sudini, Sanjay Sethi, Gerard J. Criner, Jed W. Fahey, Charles S. Berenson, Michael R. Jacobs, Rajesh Thimmulappa, Robert A. Wise, Shyam Biswal

**Affiliations:** 1 School of Medicine, Johns Hopkins University, 615 N. Wolfe St., E7622, Baltimore, MD 21205 USA; 2 Johns Hopkins Bloomberg School of Public Health, 615 N. Wolfe St., E7622, Baltimore, MD 21205 USA; 3 Lewis Katz School of Medicine at Temple University, Philadelphia, PA USA; 4 University at Buffalo, SUNY, and VA WNY Healthcare System, Buffalo, NY USA; 5 JSS Medical College, JSS Academy of Higher Education & Research, Mysuru, India

**Keywords:** COPD, Smokers, Oxidative stress, Epithelial cells, Macrophages, Nrf2

## Abstract

**Background:**

Patients with chronic obstructive pulmonary disease (COPD) have high oxidative stress associated with the severity of the disease. Nuclear factor erythroid-2 related factor 2 (Nrf2)-directed stress response plays a critical role in the protection of lung cells to oxidative stress by upregulating antioxidant genes in response to tobacco smoke. There is a critical gap in our knowledge about Nrf-2 regulated genes in active smokers and former-smokers with COPD in different cell types from of lungs and surrogate peripheral tissues.

**Methods:**

We compared the expression of Nrf2 and six of its target genes in alveolar macrophages, nasal, and bronchial epithelium and peripheral blood mononuclear cells (PBMCs) in current and former smokers with COPD. We compared cell-type specific of Nrf2 and its target genes as well as markers of oxidative and inflammatory stress.

**Results:**

We enrolled 89 patients; expression all Nrf2 target gene measured were significantly higher in the bronchial epithelium from smokers compared to non-smokers. None were elevated in alveolar macrophages and only one was elevated in each of the other compartments.

**Conclusion:**

Bronchial epithelium is the most responsive tissue for transcriptional activation of Nrf2 target genes in active smokers compared to former-smokers with COPD that correlated with oxidative stress and inflammatory markers. There were no consistent trends in gene expression in other cell types tested.

**Trial registration:**

Clinicaltrials.gov: NCT01335971.

## Background

Chronic obstructive pulmonary disease (COPD) is the third leading cause of death in the US and is primarily caused by smoking [1]. There is substantial evidence of increased oxidative stress in airways of patients with COPD, which may play a role in the development of disease and could be targeted for therapeutic benefit [2–5]. Nuclear factor erythroid-2-related factor 2 (Nrf2) is a transcription factor activated by oxidative stress, which upregulates anti-oxidant enzymes that play key roles in cellular defenses [6]. Activating Nrf2 in both mouse and human studies resulted in decreased oxidative stress and improved bacterial phagocytosis by macrophages [7–9]. The association of oxidative stress and inflammation on Nrf2 activity endogenous antioxidant defense mechanisms in different cell types in the lungs as well as surrogate tissues, such as nasal and PBMCs is not clear. Nor is it known how activity is altered in current and former-smokers with COPD. Here, we report results from the analysis of baseline data collected at the start of a randomized clinical trial to test the effectiveness of sulforaphane preparation on increasing biomarkers of Nrf2 activity. We measured levels of Nrf2 transcript and target gene levels in the nasal and lung epithelium, alveolar macrophages, and PBMCs in former and current smokers with COPD. The goal of the current analysis is to compare how current smoking alters Nrf2 activity in different cell-types in patients with COPD.

## Methods

### Study design and participants

Participants were recruited in a Phase 2, multicenter, randomized, placebo-controlled, double-masked, 3-arm parallel group trial designed to evaluate the effectiveness of oral sulforaphane on Nrf2 target gene expression and downstream anti-oxidants [10]. The focus of this manuscript is to present the data collected at the baseline visit. The clinical trial was approved by the Institutional Review Board (IRB) at each center; participants signed consent statements approved by the local IRB. The study is registered on clinicaltrials.gov and data from the trial have been deposited in the BioLINCC repository (https://biolincc.nhlbi.nih.gov/home/).

The entry criteria are described in detail elsewhere [10]. Briefly, both active and former smokers were enrolled. Participants were required to have a smoking history of 10 or more pack-years, a post-bronchodilator FEV_1_/FVC ratio < 0.70 and FEV_1_ of 40 to 80% predicted. Participants were excluded from the study for any of the following: COPD exacerbation requiring treatment within the preceding six weeks or significant co-morbidities that would interfere with study participation or interpretation of the results as described [10]. Smoking status was based on responses to the American Thoracic Society-Division of Lung Disease Respiratory Questionnaire (ATS-DLD); former smokers were those who reported not smoking in the prior month.

### Procedures

Clinical data were collected, and nasal brushings and bronchoscopy were performed at baseline. Participants provided data on medical history and COPD symptoms; completed the Saint George’s Respiratory Questionnaire (SGRQ) and the American Thoracic Society-Division of Lung Disease Respiratory Questionnaire (ATS-DLD) questionnaire and underwent a physical examination, pre- and post-bronchodilator spirometry, lung volume measurements, carbon monoxide diffusing capacity (DLCO) and pulse oximetry; and provided blood specimens. Peripheral blood mononuclear cells (PBMC) and plasma were isolated from blood. Blood was collected via venipuncture directly into two CPT tubes, mixed immediately by gently inverting the tube 8 to 10 times and then centrifuged at 1800×g for 30 min at room temperature. After centrifugation, mononuclear cells and platelets were collected with a Pasteur pipette immediately and transferred to a 50 mL falcon tube. The cells were suspended in phosphate buffer saline, mixed, and centrifuged for 8 min at 500×g and supernatant was decanted without disturbing the cell pellet. RBCs were lysed and the remaining cells numbers were counted, after which they were re-suspended in 6 mL of RPMI 1640 medium and aliquoted. The RLT lysis buffer was subsequently added to one of the pellets for gene expression analysis. The other aliquots were used for enzyme activity and functional analysis.

After performing nasal brushings to isolate nasal epithelial cells, fiberoptic bronchoscopy was performed with endobronchial brushings to collect bronchial epithelial cells and bronchoalveolar lavage to collect alveolar macrophages. Sample processing procedures were standardized across all three centers by trained laboratory personnel certified on study procedures. Specimens were collected as previously described [10]. Samples were subsequently shipped to the central laboratory for gene expression analysis. Total RNA was extracted from specimens using the RNeasy kit (Qiagen) and sample was quantifed and quality was assessed as described [10]. Gene expression was evaluated using quantitative reverse transcription real-time polymerase chain reaction (RTqPCR) as previously described [10].

We measured levels of genes in the Nrf2/Keap 1 pathway, including Nrf2, KEAP1, and SLPI as well as Nrf2 target gene expression. Specifically, the target genes we measured were NADPH, Quinone Dehydrogenase 1 (NQO1), Heme Oxygenase 1 (HO1), Aldo-Keto Reductase Family 1 Member C1 (AKR1C1), Aldo-Keto Reductase Family 1 Member C3 (AKR1C3) in alveolar macrophages, bronchial and nasal epithelial cells, as well as PBMCs. In addition, we evaluated markers of oxidative stress and inflammation including isoprostane, thiobarbituric acid reactive substances (TBARS) in plasma, and isoprostane in expired breath condensate and cytokine profiles in bronchoalveolar lavage (BAL) fluid as previously described [10]. Baseline clinical measures included spirometry and patient-reported outcomes (Medical Research Council [MRC] Dyspnea scale and St. George’s Respiratory Questionnaire [SGRQ]).

This total antioxidant capacity was measured using Cayman’s antioxidant assay kit (Cayman Chemical Company, Ann Arbor, MI, USA), which assesses the ability of the antioxidants in the sample to inhibit the oxidation of ABTS [2,2′-Azino-di-(3-ethylbenthiazoline sulphonate)] to ABTS+ by metmyoglobin. The inhibition of oxidation was compared to that of Trolox, a water-soluble tocopherol analog and the results were expressed as millimolar Trolox equivalents.

### Statistical analysis

The primary analysis was an unadjusted one-way ANOVA for fold-change in Nrf2 expression and Phase II antioxidant levels in the various cell types studied. The comparability of the participant characteristics was examined (Table 1). We quantified relative gene expression (Table 2) using the comparative cycle threshold (CT) method [11]. The expression of a target gene was quantified relative to the expression of a reference gene, β-actin, for all specimen types. Similar methods were used to evaluate all Phase II antioxidant gene expression (Table 2) and inflammatory markers (Table 3). *P*-values were adjusted for multiple comparisons using the Bonferroni adjustment,. adjusted alpha level was 0.0023 (0.05/22).

**Table 1:** Baseline characteristics by cigarette smoking

	Total	Former Smoker	Current Smoker	P-value
*N*	89	35	54	
Years of age, median (IQR)	58 (54–65)	65 (59–69)	55 (51–60)	<.001
Male, n (%)	54 (61%)	25 (71%)	29 (54%)	0.12
Race or ethnic group, *n* (%)				
White	51 (57%)	24 (69%)	27 (50%)	0.12
Black	38 (43%)	11 (31%)	26 (48%)	0.12
Hispanic	1 (1%)	0 (0%)	1 (2%)	
Smoking history median (IQR)				
Current cigarettes/day			10 (5–20)	
Age started smoking	15 (13–18)	15 (13–18)	15 (13–17)	0.34
Years since quitting	10 (4–15)	10 (4–15)		
Pack years (packs * years smoking)	39 (25–56)	39 (27–62)	39 (23–53)	0.66
COPD Characteristics *n* (%)				
COPD exacerbation, prior 12 months	19 (21%)	4 (11%)	15 (28%)	0.11
FEV1 (%predicted)	61 (53–70)	57 (53–65)	64 (53–71)	0.18
FEV1/FVC ratio	0.56 (0.48–0.62)	0.53 (0.46–0.59)	0.57 (0.50–0.63)	0.09
DLCO (mL/mm/mmHg)	15.7 (12.1–20.9)	16.1 (11.9–21.2)	15.7 (12.1–20.9)	0.95
TLC (Liters)	6.0 (5.0–7.2)	6.3 (5.3–7.4)	5.7 (5.0–7.0)	0.2
SVC (Liters)	3.3 (2.7–4.1)	3.7 (2.7–4.2)	3.2 (2.6–4.0)	0.49
FRC (Liters)	3.5 (3.0–4.2)	3.6 (3.0–4.1)	3.5 (3.0–4.2)	0.76
RV (Liters)	2.6 (2.2–3.2)	2.7 (2.4–3.5)	2.6 (1.9–3.0)	0.09
Pulse oximetry (%)	96 (94–97)	95 (93–97)	96 (95–98)	0.01
Short acting beta-agonist (SABA)	61 (69%)	22 (63%)	39 (72%)	0.36
LABA and inhaled corticosteroid	40 (45%)	17 (49%)	23 (43%)	0.66
Long-acting anticholinergic	26 (29%)	11 (31%)	15 (28%)	0.81
Aspirin	22 (25%)	14 (40%)	8 (15%)	0.01
Questionnairre scores (median (IQR)				
Medical Research Council Dyspnea	2 (1–3)	2 (1–3)	2 (1–3)	0.41
St George’s Respiratory	40 (26–56)	39 (26–56)	46 (26–62)	0.42

Abbreviations: *FEV1* Forced expired volume in 1 s, *FVC* Forced vital capacity, *DLCO* Diffusing capacity of the lungs for carbon monoxide, *TLC* Total lung capacity, *SVC* Slow vital capacity, *FRC* Functional residual volume, *RV* Residual volume

**Table 2:** Baseline measures of genetic expression (fold-change) by cigarette smoking status

	Former Smoker *N* = 34	Current Smoker *N* = 53	*P*-value*
	Median (IQR)		
Bronchial epithelial cells			
NRF2	0.83 (0.65–1.17)	1.03 (0.91–1.26)	0.010
NQO1	0.56 (0.36–0.75)	1.61 (1.01–2.22)	<.001
HO1	0.64 (0.31–0.92)	1.29 (0.61–1.87)	<.001
AKR1C1	0.58 (0.31–0.94)	1.72 (0.76–2.64)	<.001
AKR1C3	0.64 (0.36–1.05)	1.54 (0.98–2.34)	<.001
Keap1	0.97 (0.64–1.25)	1.16 (0.89–1.53)	0.03
Alveolar macrophages			
NRF2	1.32 (0.94–1.72)	1.05 (0.77–1.42)	0.05
NQO1	0.65 (0.47–1.70)	1.11 (0.62–2.05)	0.18
HO1	1.12 (0.76–1.44)	0.99 (0.74–1.25)	0.33
AKR1C1	1.34 (0.64–4.49)	0.88 (0.57–2.88)	0.20
AKR1C3	0.97 (0.53–1.48)	1.11 (0.85–1.68)	0.29
Keap1	0.91 (0.70–1.44)	0.99 (0.79–1.32)	0.83
Nasal epithelial cells			
NRF2	1.00 (0.80–1.13)	1.10 (0.78–1.41)	0.13
NQO1	1.05 (0.59–1.80)	1.00 (0.56–2.05)	0.92
AKR1C3	1.21 (0.67–1.97)	1.13 (0.69–2.46)	0.79
AKR1B10	0.67 (0.39–1.59)	2.19 (0.99–4.43)	0.001
PBMC			
NRF2	0.85 (0.69–1.04)	1.05 (0.82–1.43)	0.009
NQO1	0.85 (0.53–1.04)	0.92 (0.64–1.25)	0.30
HO1	1.43 (1.12–1.68)	1.07 (0.84–1.37)	0.003
AKR1C1	1.82 (0.40–6.73)	0.73 (0.37–8.88)	0.82
AKR1C3	0.72 (0.51–1.16)	0.89 (0.51–1.13)	0.61

**Table 3:** Baseline measures of antioxidants and markers of inflammation by smoking status

	Former Smoker	Current Smoker	*P*-value
	Median (Interquartile Range)		
Serum (*N*)	34	53	
C-reactive protein (mg/L)	8.3 (3.0–11.6)	6.0 (2.6–13.6)	0.86
Interleukin-6 (pg/mL)	2.0 (1.3–3.5)	2.2 (1.3–3.2)	0.83
Interleukin-8 (pg/mL)	10.1 (7.2–13.5)	13.2 (10.0–17.2)	0.03
Bronchial Alveolar Lavage (N)	33	50	
Interleukin-8 (pg/mg)	1.7 (0.8–3.4)	2.6 (1.0–5.1)	0.09
SLPI (pg/mg)	283 (228–469)	337 (203–455)	0.79
Expired Breath Condensate (*N*)	32	53	
Isoprostane (ng/mg)	27.4 (6.9–49.9)	12.1 (6.9–26.6)	0.08
Plasma (*N*)	34	53	
Isoprostane (ng/mg)	144 (45–223)	230 (94–512)	0.02
TBARS (nmol MDA/mL)	8.1 (6.1–8.9)	7.4 (5.8–8.8)	0.55
Total antioxidants (mM Trolox equivalents/L)	0.65 (0.59–0.70)	0.62 (0.54–0.66)	0.03

Abbreviations: *TBARS* Thiobarbituric acid reactive substances; *SLPI* Secretory leukoprotease inhibitor. Statistical analysis performed using one-way Anova with Bonferroni correction for multiple comparators

For the comparison of former and current smokers by cell type the median of all measurements within cell type was used as the calibrator. Non-parametric tests (Wilcoxon rank-sum tests) were used to assess differences in fold-change in Nrf2 expression (Table 2) and Phase II antioxidant levels (Table 3) between former and current smokers in the various cell types studied. For the comparison of expression levels across all cell types the median of all measurements was used as the calibrator. Differences in expression between cells types were evaluated by the paired Wilcoxon signed-rank test. Data were analyzed using SAS (version 9.3).

### Role of the funding source

This study was funded by NIH/NHLBI (Grant Number U01HL105569). The sponsor had no role in study design, data collection, data analysis, data interpretation or writing of the report.

## Results

We analyzed the baseline characteristics of the eighty-nine participants enrolled in the trial (Table 1). Sixty-one percent of participants (54/89) were current smokers at the time of enrollment. Former smokers (35/89) had quit smoking for a median of 10 years (4–15 IQR) at a median age of 56 years (47–61 IQR). Both groups had the same median exposure to cigarette smoke, 39 pack-years (25–56 IQR). Former smokers were older; and were more likely to report hypertension, otherwise, demographics and COPD characteristics were similar between the former and current smokers. 57% of the participants were white and 43% were black and none were on supplemental oxygen. The majority of the participants (61%) had used a short-acting beta-agonist in the previous two weeks.

There was no statistically significant difference in the number of participants that reported a COPD exacerbation in the prior 12 months; 11% of the former smokers and 28% of the current smokers had a COPD exacerbation.

**Fig. 1:**
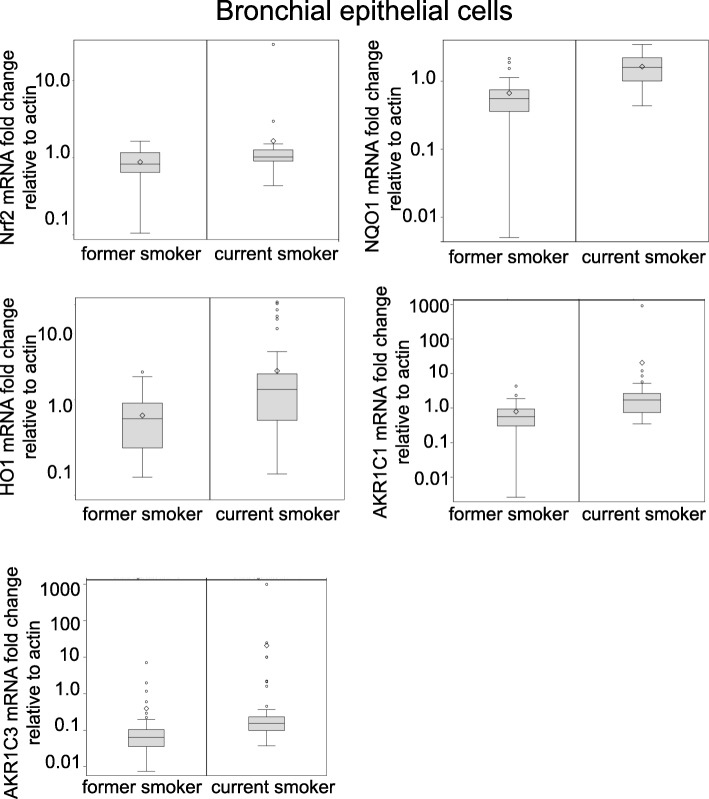
Association of tobacco use with Nrf2 target gene transcription. In the bronchial epithelium there was no difference in Nrf2 expression by smoking status (*P* = 0.01), but there was higher transcriptional expression of the downstream Nrf2 gene expression in NQO1 (*p* < 0.001), HO1 (*p* < 0.001), HO1 (*p* < 0.001), AKR1C1 (*p* < 0.001), and AKR1C3 (*p* < 0.001), which were statistically significant even with the Bonferroni adjusted alpha level of 0.0023 (0.05/22) to account for multiple comparisons

A series of Nrf2 target genes were studied. There was a statistically different expression in the bronchial epithelial cells between former and current tobacco users in the entire series of target genes (Table 2, Fig. 1), although Nrf2 gene expression was not statistically higher in the bronchial epithelial cells obtained from brushing from active smokers compared to that in former smokers (1.03 vs 0.83, *p* = 0.01). Keap 1 levels also were not increased in the bronchial epithelial cells of active smokers (*p* = 0.03).

There were no statistically significant differences in the levels of most Nrf2 target genes measured in the other cell types studied with one exception (Table 2). In nasal epithelium there was a statistically significant higher levels of AKR1B10 expression in current smokers (2.19 vs 0.67, *p* = 0.001) and a decrease in SLPI in the nasal epithelium of current smoker (0.69 vs 1.46, *p* < 0.001, not shown).

Since former smokers were older than current smokers (Table 1), we examined the effects of age by comparing expression in participants older than age 57 years vs. younger participants. Only NQO1 gene expression in bronchial epithelium was related to age (median expression 0.72 in older vs 1.58 in younger participants). Expression for NQO1 in bronchial epithelium was examined stratified by age group;in both age groups expression was higher in current smokers than former smokers.

There were no identifiable differences antioxidant or inflammatory markers measured in the serum, plasma, bronchoalveolar lavage, or expired breath condensate between former and current smokers (Table 3). When specific components such as isoprostane and TBARS were compared, the associations were less robust due to the skewed distributions (data not shown). The numbers of subjects were not high enough to perform correlations between antioxidant levels and number of cigarettes used in the current smoker group.

**Fig. 2:**
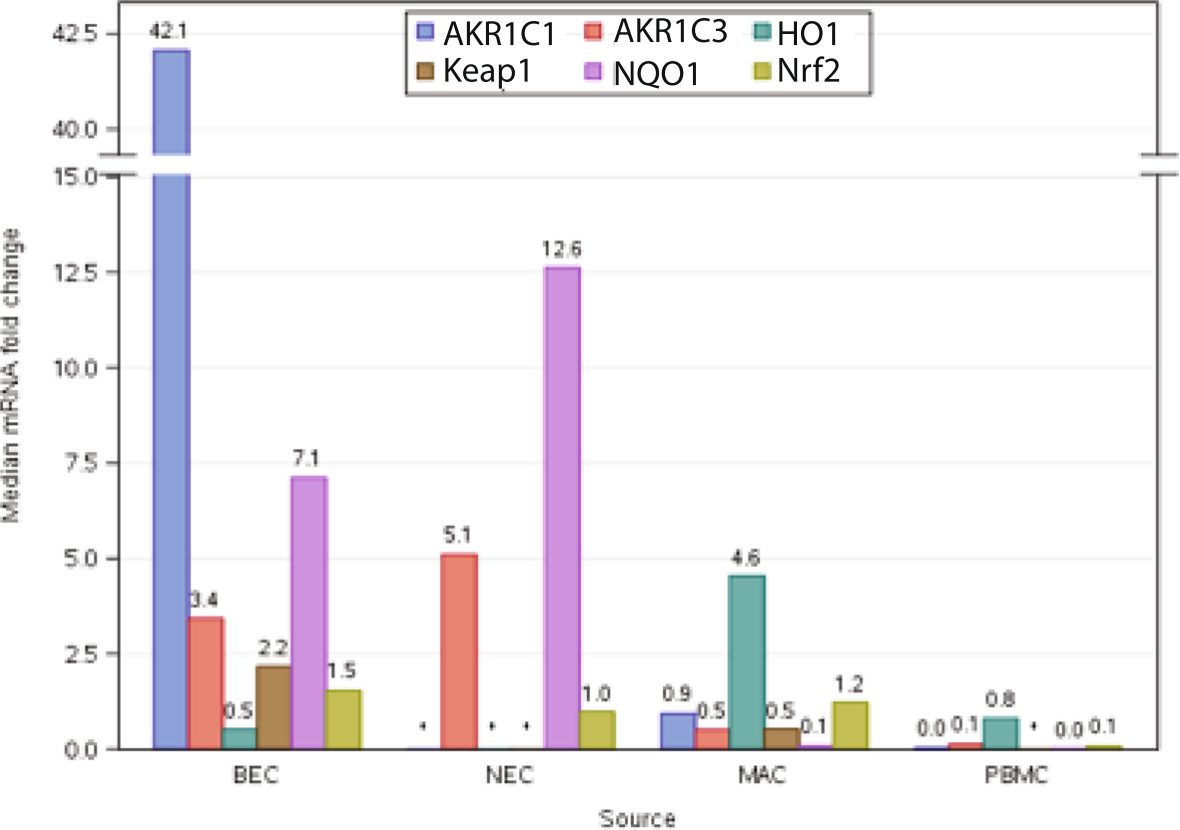
mRNA levels of each target gene from each source. All pairwise comparisons of target gene transcript across the different cell types indicated significant (*p* < .0001) differences between sources except for Nrf2 expression in bronchial epithelium vs macrophages (*p* = 0.01); AKR1C3 in bronchial epithelium vs nasal epithelium (*p* = 0.74); and HO1 in bronchial epithelium vs peripheral blood mononuclear cells (*p* = 0.09)

We compared Nrf2 transcript levels in the various cell types. When analyzing all participants, levels of Nrf2 expression was higher in the lung (both bronchial epithelium and alveolar macrophages) compared to PBMCs and nasal epithelium (Fig. 2). Expression of most of the target gene measured was significantly elevated in the bronchial epithelium compared to alveolar macrophages and PBMCs with the exception of HO1, which was higher in those tissues. The full panel of target genes could not be measured in the nasal epithelium, but levels of NQO1 and AKR1C3 expression were significantly higher than even bronchial epithelium. Interestingly, active smoking status did not influence the trends in expression. An individual’s expression was generally not correlated across source for any of the Nrf2 target genes using pairwise comparisons with the exception of negative correlations in HO1 in bronchial epithelium compared to PBMC (*p* = <.0001,Spearman rank correlation = − 0.46).

## Discussion

Nrf2 is a key modifier of responses against oxidative stress and inflammation [8, 12–15]. Nrf2 disruption in murine models causes early onset changes in lung architecture and more severe emphysema after chronic cigarette smoke exposure, and is associated with oxidative stress, inflammation, and apoptosis of type II and endothelial lung cells [16, 17]. Transcriptional targets of Nrf2 are well-defined antioxidant and detoxification genes [18]. In healthy smokers, Nrf2 target genes are upregulated [19, 20], however there is a decrease in Nrf2 activity with the progression of COPD [21]. How active smoking modifies Nrf2 activity in patients with COPD in various cell types has not been well studied. Because it is easier to obtain PBMCs it is tempting to extrapolate those findings to other cellular compartments in the lung. Our current study demonstrates that this may not be warranted. Also, the gene expression profiles in alveolar macrophages may differ from those of the central airway epithelium.

There was no statstically significant differences in Nrf2 mRNA levels by smoking status, which is not surprising as Nrf2 is typically regulated at a protein level. However, we showed that there is a distinct difference in the levels of Nrf2-related transcripts primarily in bronchial epithelium in former versus current smoking status in patients with COPD. Therefore, while Nrf2 activity may be lower with COPD as previously suggested [21, 22], this level can be upregulated with active smoking, presumably due to the ongoing oxidative stress and inflammation with active smoking.

The finding of increased Nfr2 activity in the bronchial epithelium is consistent with increases in small airway epithelium of healthy smokers found by Carolan et al. [19]. Although there has been more focus on the epithelium of the small airways [19, 23–25], there is increasing evidence of chronic changes in the large airway epithelium as well [26–28], which our study supports. It is reasonable to presume that upregulation in Nrf2 activity is a response to local exposure, and it explains why peripheral blood cells may not reflect similar changes. This is particularly of significance because due to the ease of sampling, investigators often substitute peripheral immune effector cells to study changes that may, in fact, be compartmentalized to the lung epithelium.

It is noteworthy that active smoking does not result in an increase in Nrf2 activity in alveolar macrophages isolated by bronchoalveolar lavage, indicating that immune effector cell responses to smoke may be subdued compared to epithelial responses. This is in contrast to bronchial epithelial cells, where Nrf2 target gene activity is higher than in macrophages in COPD patients with former tobacco use for most target genes and further increased with active smoking. Alveolar macrophages from smokers have been shown to have defects in phagocytosis [8, 9, 29–31] that are Nrf2 dependent, and therefore macrophage oxidative stress has been of great interest. There was no association between smoking status and Nrf2 activity in macrophages, which may reflect relative resistance to chronic tobacco smoke as a regulator of Nrf2 activity. Alternatively, macrophages do not accumulate oxidative stress over time, which is reflected by a weaker Nrf2 signal. Further mechanistic studies are needed to address these questions. As Nrf2 activity in normal controls was not measured in this study, we cannot comment on the relative change in Nrf2 activity in the alveolar macrophages in COPD patients, although others have found a decrease in Nrf2 levels in COPD [19, 32]. However, it does not appear to be further influenced by active smoking.

There are some important limitations to note. First, the number of participants studied was relatively small with only 89 subjects. The definition of non smoking required only one month’s abstinence because we relied on the ATS-DLD questionnaire responses. There were also higher levels of aspirin use in former smokers than current smokers but the anti-oxidants effects of aspirin are controversial with some studies suggesting that it may have some anti-oxidant properties [33, 34] while others indicate that it actually impairs the anti-oxidant system [35]. There are some recent studies that suggest that aspirin may modulate Nrf2 activity [36–38], however these are at micromolar concentrations. A 325 mg tablet of aspirin would result in approximately 2.2 nM concentrations in the blood, assuming complete absorption and equal distributions in all tissues, and there is no evidence that these low concentrations would influence Nrf2 activity. Regardless, larger sample sizes are needed to evaluate potential effects of aspirin. There was no consistent differences in use of other medications identifed that would confound the findings, aside from what was reported in Table 1. In addition, the relatively small numbers of patients would preclude us from determining if any specific co-morbidities influenced the findings.

## Conclusion

Our study is the first assessment of Nrf2 levels and activity in different cellular compartments in response to active tobacco use in patients with COPD. Recognizing the differences in levels of Nrf2 between different compartments will allow us to determine whether specific cellular targeting is required to have the therapeutic efficacy of Nrf2 activators as has been seen in animal and in vitro studies. Accordingly, caution is warranted in extrapolating smoking-related gene expression from other cellular compartments to the central airway epithelium.

## Data Availability

Data from the trial has been deposited in the BioLINCC repository as stated above.

## Abbreviations

AKR1B10: Aldo-Keto Reductase Family 1 Member B10
AKR1C1: Aldo-Keto Reductase Family 1 Member C1
AKR1C3: Aldo-Keto Reductase Family 1 Member C3
COPD: chronic obstructive pulmonary disease
FEV_1_: Forced expiratory volume in 1 s
FVC: Forced vital capacity
HO1: Heme Oxygenase 1
IQR: Interquartile range
KEAP1: Kelch-like ECH-associated protein 1
NADPH: Reduced nicotinamide adenine dinucleotide phosphate
NQO1: Quinone Dehydrogenase 1
Nrf2: Nuclear factor erythroid-2 related factor 2
PBMC: peripheral blood mononuclear cells
RBC: Red blood cells
RLT lysis buffer: RNA lysis buffer
RPMI: Roswell Park Memorial Institute media
SLPI: Secretory Leukocyte Peptidase Inhibitor
TBARS: Thiobarbituric acid reactive substances

## References

[1] Barnes PJ, Shapiro SD, Pauwels RA (2003). Chronic obstructive pulmonary disease: molecular and cellular mechanisms. Eur Respir J.

[2] Kirkham PA, Barnes PJ (2013). Oxidative stress in COPD. Chest..

[3] Rahman I (2005). The role of oxidative stress in the pathogenesis of COPD: implications for therapy. Treat Respir Med.

[4] van Eeden SF, Sin DD (2013). Oxidative stress in chronic obstructive pulmonary disease: a lung and systemic process. Can Respir J.

[5] Repine JE, Bast A, Lankhorst I (1997). Oxidative stress in chronic obstructive pulmonary disease. Oxidative stress study group. Am J Respir Crit Care Med.

[6] Ma Q (2013). Role of nrf2 in oxidative stress and toxicity. Annu Rev Pharmacol Toxicol.

[7] Biswal S, Thimmulappa RK, Harvey CJ (2012). Experimental therapeutics of Nrf2 as a target for prevention of bacterial exacerbations in COPD. Proc Am Thorac Soc.

[8] Harvey C. J., Thimmulappa R. K., Sethi S., Kong X., Yarmus L., Brown R. H., Feller-Kopman D., Wise R., Biswal S. (2011). Targeting Nrf2 Signaling Improves Bacterial Clearance by Alveolar Macrophages in Patients with COPD and in a Mouse Model. Science Translational Medicine.

[9] Berenson CS, Garlipp MA, Grove LJ, Maloney J, Sethi S (2006). Impaired phagocytosis of nontypeable Haemophilus influenzae by human alveolar macrophages in chronic obstructive pulmonary disease. J Infect Dis.

[10] Wise RA, Holbrook JT, Criner G, Sethi S, Rayapudi S, Sudini KR (2016). Lack of effect of Oral Sulforaphane administration on Nrf2 expression in COPD: a randomized, double-blind. Placebo Controlled Trial PloS one.

[11] Livak KJ, Schmittgen TD (2001). Analysis of relative gene expression data using real-time quantitative PCR and the 2(−Delta Delta C(T)) method. Methods..

[12] Osburn WO, Kensler TW (2008). Nrf2 signaling: an adaptive response pathway for protection against environmental toxic insults. Mutat Res.

[13] Kensler TW, Wakabayashi N, Biswal S (2007). Cell survival responses to environmental stresses via the Keap1-Nrf2-ARE pathway. Annu Rev Pharmacol Toxicol.

[14] Yan J, Li J, Zhang L, Sun Y, Jiang J, Huang Y (2018). Nrf2 protects against acute lung injury and inflammation by modulating TLR4 and Akt signaling. Free Radic Biol Med.

[15] Lugade AA, Vethanayagam RR, Nasirikenari M, Bogner PN, Segal BH, Thanavala Y (2011). Nrf2 regulates chronic lung inflammation and B-cell responses to nontypeable Haemophilus influenzae. Am J Respir Cell Mol Biol.

[16] Rangasamy T, Misra V, Zhen L, Tankersley CG, Tuder RM, Biswal S (2009). Cigarette smoke-induced emphysema in a/J mice is associated with pulmonary oxidative stress, apoptosis of lung cells, and global alterations in gene expression. Am J Physiol Lung Cell Mol Physiol.

[17] Rangasamy T, Cho CY, Thimmulappa RK, Zhen L, Srisuma SS, Kensler TW (2004). Genetic ablation of Nrf2 enhances susceptibility to cigarette smoke-induced emphysema in mice. J Clin Invest.

[18] McMahon M, Itoh K, Yamamoto M, Chanas SA, Henderson CJ, McLellan LI (2001). The Cap'n'Collar basic leucine zipper transcription factor Nrf2 (NF-E2 p45-related factor 2) controls both constitutive and inducible expression of intestinal detoxification and glutathione biosynthetic enzymes. Cancer Res.

[19] Carolan BJ, Harvey BG, Hackett NR, O'Connor TP, Cassano PA, Crystal RG (2009). Disparate oxidant gene expression of airway epithelium compared to alveolar macrophages in smokers. Respir Res.

[20] Hubner RH, Schwartz JD, De Bishnu P, Ferris B, Omberg L, Mezey JG (2009). Coordinate control of expression of Nrf2-modulated genes in the human small airway epithelium is highly responsive to cigarette smoking. Mol Med.

[21] Goven D, Boutten A, Lecon-Malas V, Marchal-Somme J, Amara N, Crestani B (2008). Altered Nrf2/Keap1-Bach1 equilibrium in pulmonary emphysema. Thorax..

[22] Boutten A, Goven D, Boczkowski J, Bonay M (2010). Oxidative stress targets in pulmonary emphysema: focus on the Nrf2 pathway. Expert Opin Ther Targets.

[23] Strulovici-Barel Y, Omberg L, O'Mahony M, Gordon C, Hollmann C, Tilley AE (2010). Threshold of biologic responses of the small airway epithelium to low levels of tobacco smoke. Am J Respir Crit Care Med.

[24] Walters MS, De BP, Salit J, Buro-Auriemma LJ, Wilson T, Rogalski AM (2014). Smoking accelerates aging of the small airway epithelium. Respir Res.

[25] Sohal SS, Walters EH (2013). Epithelial mesenchymal transition (EMT) in small airways of COPD patients. Thorax..

[26] Gohy ST, Hupin C, Fregimilicka C, Detry BR, Bouzin C, Gaide Chevronay H (2015). Imprinting of the COPD airway epithelium for dedifferentiation and mesenchymal transition. Eur Respir J.

[27] Sohal SS, Walters EH (2013). Role of epithelial mesenchymal transition (EMT) in chronic obstructive pulmonary disease (COPD). Respir Res.

[28] Sohal SS, Reid D, Soltani A, Weston S, Muller HK, Wood-Baker R (2013). Changes in airway histone deacetylase2 in smokers and COPD with inhaled corticosteroids: a randomized controlled trial. PLoS One.

[29] Berenson CS, Wrona CT, Grove LJ, Maloney J, Garlipp MA, Wallace PK (2006). Impaired alveolar macrophage response to Haemophilus antigens in chronic obstructive lung disease. Am J Respir Crit Care Med.

[30] Hiemstra PS (2013). Altered macrophage function in chronic obstructive pulmonary disease. Ann Am Thorac Soc.

[31] Bewley MA, Budd RC, Ryan E, Cole J, Collini P, Marshall J (2018). Opsonic phagocytosis in chronic obstructive pulmonary disease is enhanced by Nrf2 agonists. Am J Respir Crit Care Med.

[32] Suzuki M, Betsuyaku T, Ito Y, Nagai K, Nasuhara Y, Kaga K (2008). Down-regulated NF-E2-related factor 2 in pulmonary macrophages of aged smokers and patients with chronic obstructive pulmonary disease. Am J Respir Cell Mol Biol.

[33] Blatter-Garin MC, Kalix B, De Pree S, James RW (2003). Aspirin use is associated with higher serum concentrations of the anti-oxidant enzyme, paraoxonase-1. Diabetologia..

[34] Grosser N, Abate A, Oberle S, Vreman HJ, Dennery PA, Becker JC (2003). Heme oxygenase-1 induction may explain the antioxidant profile of aspirin. Biochem Biophys Res Commun.

[35] Durak I, Karaayvaz M, Cimen MY, Avci A, Cimen OB, Buyukkocak S (2001). Aspirin impairs antioxidant system and causes peroxidation in human erythrocytes and Guinea pig myocardial tissue. Hum Exp Toxicol.

[36] Huang MZ, Yang YJ, Liu XW, Qin Z, Li JY (2019). Aspirin eugenol ester attenuates oxidative injury of vascular endothelial cells by regulating NOS and Nrf2 signalling pathways. Br J Pharmacol.

[37] Wei W, Shurui C, Zipeng Z, Hongliang D, Hongyu W, Yuanlong L (2018). Aspirin suppresses neuronal apoptosis, reduces tissue inflammation, and restrains astrocyte activation by activating the Nrf2/HO-1 signaling pathway. Neuroreport..

[38] Jian Z, Tang L, Yi X, Liu B, Zhang Q, Zhu G (2016). Aspirin induces Nrf2-mediated transcriptional activation of haem oxygenase-1 in protection of human melanocytes from H2 O2 -induced oxidative stress. J Cell Mol Med.

